# TRANSFORMING AN INTERGENERATIONAL SERVICE-LEARNING PROJECT DURING THE COVID-19 PANDEMIC

**DOI:** 10.1093/geroni/igac059.1531

**Published:** 2022-12-20

**Authors:** Renee' Zucchero, Annaliet Delgado-Rodriguez

**Affiliations:** Xavier University, Cincinnati, Ohio, United States; Xavier University, Cincinnati, Ohio, United States

## Abstract

The COVID-19 pandemic has required transformation in the delivery of higher education and pedagogy that is used. The Co-Mentoring Project links undergraduate Psychology of Aging students with older adult volunteers for an intergenerational service-learning experience. Prior to the pandemic, the Project was delivered via an in-person format. During the pandemic, the Project transitioned to a virtual format. Self-reported postproject evaluations from undergraduate students (n= 30) and older adults (n= 27) during the two academic years prior to the pandemic were compared to evaluations from students (n= 26) and older adults (n= 28) during two years of the pandemic. Mann-Whitney U Tests revealed no significant differences in older adult and student postproject evaluation outcomes between in-person and virtual formats. For example, there were no differences in older adult level of enjoyment between the in-person (Md= 5, n= 27) and virtual formats (Md= 5, n= 28), U = 405.00, z = .84, p = .40. Likewise, there no differences in student level of comfort interacting with older adults between in-person (Md= 5, n= 30) and virtual formats (Md= 5, n= 26), U = 389.00, z = -.02, p = .99. Qualitative information from the postproject evaluations indicated participants were glad to have had the opportunity to meet virtually during the pandemic, however they preferred an in-person format. These results support the conclusion that intergenerational service-learning can be successfully implemented virtually. This paper will describe the transformation of the Project from an in-person to virtual format, and advantages and disadvantages of both formats.

